# Genetic Diversity of Clinical *Bordetella Pertussis* ST2 Strains in comparison with Vaccine Reference Strains of India

**DOI:** 10.7150/jgen.58823

**Published:** 2021-09-03

**Authors:** Naresh Chand Sharma, Shalini Anandan, Naveen Kumar Devanga Ragupathi, Dhiviya Prabaa Muthuirulandi Sethuvel, Karthick Vasudevan, Dhirendra Kumar, Sushil Kumar Gupta, Lucky Sangal, Balaji Veeraraghavan

**Affiliations:** 1 Maharishi Valmiki Infectious Diseases Hospital, New Delhi - 110009, India.; 2 Department of Clinical Microbiology, Christian Medical College, Vellore - 632 004, India.; 3 Department of Chemical and Biological Engineering, The University of Sheffield, Sheffield, United Kingdom.; 4 World Health Organization, Country Office, New Delhi - 110029, India.

**Keywords:** *Bordetella pertussis*, * ptx*, IS*481*, genome reduction, ST2, Hybrid assembly

## Abstract

**Objectives:** Pertussis is a highly contagious disease of the respiratory tract caused by *Bordetella pertussis*, a bacterium that lives in the mouth, nose, and throat. Current study reports the highly accurate complete genomes of two clinical *B. pertussis* strains from India for the first time.

**Methods:** Complete genome sequencing was performed for two *B. pertussis* strains using Ion Torrent PGM and Oxford nanopore sequencing method. Data was assembled *de novo* and the sequence annotation was performed through PATRIC and NCBI server. Downstream analyses of the isolates were performed using CGE server databases for antimicrobial resistance genes, plasmids, and sequence types. The phylogenetic analysis was performed using Roary.

**Results:** The analysis revealed insertional elements flanked by IS*481*, which has been previously regarded as the important component for bacterial evolution. The two *B. pertussis* clinical strains exhibited diversity through genome degradation when compared to whole-cell vaccine reference strains of India. These isolates harboured multiple genetic virulence traits and toxin subunits, which belonged to sequence type ST2.

**Conclusion:** The genome information of Indian clinical *B. pertussis* strains will serve as a baseline data to decipher more information on the genome evolution, virulence factors and their role in pathogenesis for effective vaccine strategies.

## Introduction

Pertussis, caused by *Bordetella pertussis,* is a highly contagious respiratory infection, characterized by severe episodes of coughing and a prolonged convalescent period when the patient can transmit the disease 1. Although large scale vaccination reduced the incidence of the disease, recent trends suggest a re-emergence particularly among the adolescent and young adult population in the developed countries. The reason for this resurgence is uncertain, apart from waning immunity which needs to be studied further. Further, the impact of vaccination needs to be established since more than one strain used in the manufacturing of pertussis vaccines globally. The comparative genomic analysis would provide high resolution data to study the structural variations in the circulating strains of *B. pertussis* against vaccine strains.

However, genome data of *B. pertussis* is rare in India due to the complexity of culturing* B. pertussis* from clinical samples. Recent studies revealed the genomic structural heterogeneity among the isolates within the geographical or temporally defined epidemics 2. These studies illustrated that genome evolution in *B. pertussis* is mainly due to rearrangement in addition to genome reduction 2. Genomic variations are mostly studied in the circulating strains of *B. pertussis* to know the pathogen adaptation to the vaccine antigens such as pertussis toxin (*ptx*), pertactin (*prn*), fimbriae (*fim*) and filamentous hemagglutinin (FHA) 3. Understanding the molecular epidemiology of this pathogen is essential which will be helpful for facilitating public heath surveillance in the country. Here we report the comparative genomic analysis of two clinical isolates of *B. pertussis* from India against the vaccine reference strains 6229, 25525, 134, 509, 10536 and Tohama I.

## Materials and Methods

### Strain Isolation and Characterization

Bacterial strains BPD1 and BPD2 isolated from nasopharyngeal swabs of paediatric patients in the year 2017 as a part of surveillance study funded by World Health Organization, India. Swabs were plated in charcoal blood agar and incubated at 37 °C with CO2 for 48 hours. Isolates were confirmed by standard biochemical tests and real-time PCR for the targets IS*481* and *ptxS1* genes 4.

### Genome sequencing

#### Short read sequencing and assembly

Genomic DNA of the *B. pertussis* isolates were extracted using QIAamp DNA Mini Kit (QIAGEN, Hilden, Germany). Whole genome sequencing (WGS) was performed in IonTorrent^TM^ Personal Genome Machine^TM^ (PGM) (Life Technologies, Carlsbad, CA) with 400-bp read chemistry as per manufacturer's instructions. Raw reads were assembled *de novo* using Assembler SPAdes v.5.0.0.0 embedded in Torrent Suite Server v.5.0.3.

#### Long read sequencing and assembly

Library preparation and sequencing of the *B. pertussis* isolates was done using SQK-LSK108 Kit R9 version (Oxford Nanopore Technologies, Oxford, UK) using 1D sequencing method according to manufacturer's protocol. Sequencing of the isolates was performed using FLO-MIN106 R9 flow cell in MinION Mk 1B sequencer. Briefly, genomic DNA was purified using 1X AMPure beads (Agencourt, Beckman Coulter, Brea CA, USA). Purified DNA was subjected to dA-tailing using NEBNext dA-Tailing Module (New England Biolabs), followed by ligation with the leader and hairpin sequencing adapters (Oxford Nanopore Technologies, Oxford, UK) using Blunt TA Ligase master mix (New England Biolabs). Barcode adapters were ligated using BAM 1D (Oxford Nanopore Technologies) followed by AMPure beads purification and elution in 15 μL of Elution Buffer (Oxford Nanopore Technologies, Oxford, UK). Prepared DNA was then loaded onto the flow cell.

To perform sequencing, MinKNOW software ver. 1.15.1 (Oxford Nanopore Technologies, Oxford, UK) was used in a Windows platform and raw data (fast5 files) were obtained. The Fast5 files were basecalled with Albacore 2.0.1 (https://nanoporetech.com/about-us/news/new-basecaller-now-performs-raw-basecalling-improved-sequencing-accuracy). Error correction and genome assembly was performed using Canu 1.7 5. The obtained contigs were polished with Nanopolish 0.10.1 (https://github.com/jts/nanopolish) after *de novo* assembly.

#### Hybrid assembly using IonTorrent and MinION reads

Hybrid assembly using both IonTorrent and MinION reads were performed to increase the accuracy and completeness of genome. Unicycler (v0.4.6) was used for generating hybrid assemblies 6. Further, the reads were polished with multiple rounds of Pilon v1.24 7 to reduce the base level errors. The assembly statistics and average nucleotide identity of different assemblies were evaluated using Quast v5.0.2 8.

### Genome annotation and MLST analysis

Annotation of the sequences were done using PATRIC, the bacterial bioinformatics database and analysis resource (http://www.patricbrc.org) 9, and NCBI Prokaryotic Genomes Automatic Annotation Pipeline (PGAAP, http://www.ncbi.nlm.nih.gov/genomes/static/Pipeline.html). Taxonomy identification was performed using PathogenFinder 1.1 (https://cge.cbs.dtu.dk/services/PathogenFinder/).

Virulence genes and antibiotic resistance genes were identified using VFDB database (http://www.mgc.ac.cn/cgi-bin/VFs/v5/main.cgi) and ResFinder 4.0 (https://cge.cbs.dtu.dk/services/ResFinder/). Mobile genetic elements (MGEs) like prophages, CRISPR, and Genomic islands were analysed using PHASTER, CRISPR Finder, and Island viewer 10-12. Plasmids were identified using PlasmidFinder 2.1 (https://cge.cbs.dtu.dk/services/PlasmidFinder/). MLST 1.8 (Multi Locus Sequence Typing) tool was employed for sequence type analysis (https://cge.cbs.dtu.dk//services/MLST/) 13.

### Phylogenomic analysis

The clinical strains BPD1 and BPD2 were compared with the previously reported vaccine reference strains 6229 (CP017404), 25525 (CP017405), 134 (CP017402), 509 (CP017403), 10536 (CP012228) and Tohama I (NC_002929). The phylogenetic analysis was performed using Roary: The Pan Genome Pipeline from Sanger Institute v3.11.2 14. Phylogenetic tree was constructed using Interactive Tree of Life (iTOL) v3 15.

## Results and Discussion

### Genome length, CDS and ST types

Hybrid assemblies returned with 203X and 195X coverage for BPD1 and BPD2 isolates respectively for the complete genomes in MinION platform. The completed BPD1 genome had a sequence length of 4,126,211 bp with 3941 CDS, 3 rRNA and 50 tRNA, and isolate BPD2 had 4,104,911 bp with 3921 CDS, 3 rRNA and 51 tRNA (https://www.patricbrc.org). The isolates did not return any antimicrobial resistance genes or plasmids as analysed by ResFinder and PlasmidFinder. The whole genome MLST revealed the sequence type (ST) of both isolates to be ST2 belonging to CC2, previously reported to be unique to Africa 16.

### Insertional elements observed in vaccine reference strains

**Figure 1:**
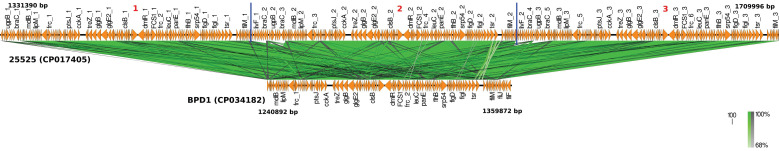
Representation of similarity and internal inverted repeats in comparison of repeat region flanked by IS*481* from BPD1 (CP034182) and the vaccine reference strain 25525 (CP017405). Three copies of same region were found in 25525, whereas only one copy was identified in BPD1.

**Figure 2:**
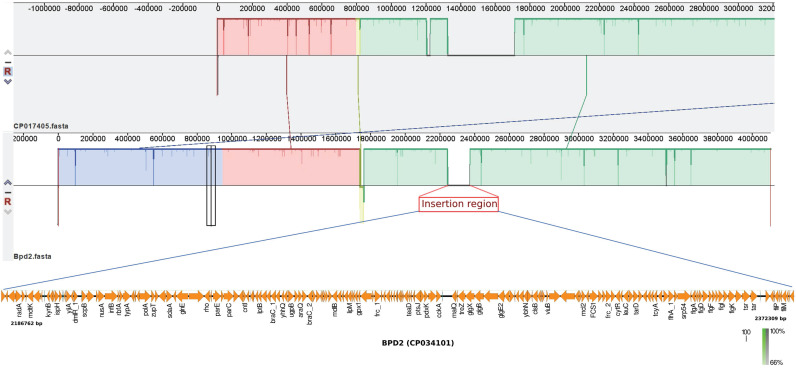
Comparison of BPD2 (CP034101) and vaccine strain 25525 (CP017405) revealed the presence of insertion with repeat region flanked by IS*481* in BPD2 which is absent in the vaccine strain.

Previous study suggests that the adaptation of *B. pertussis* to the human population has been associated with considerable gene loss and gene inactivation due to insertion sequence (IS) element expansion and mutations, a process commonly seen in host-restricted bacteria 17. In this study, BPD1 had included ~120 Kb repeat insertion flanked by copies of IS*481* in single copy (Table 1), while, BPD2 had an insertion of ~180 Kb length. However, these isolates lacked other repeat regions that were observed in the vaccine reference strains 6229 (CP017404) and 25525 (CP017405) used for production of whole-cell vaccine (WCV) in India belonging to ST2 2. Comparison of the repeat region observed in BPD1 with 25525 reference strain using Easyfig v2.2.3 showed the similarity between the two regions with internally inverted repeat regions which is absent in BPD2 (Figure 1). In addition, three copies of same region were found in 25525 while only one copy was found in BPD1 which mainly contains flagellar genes, transcriptional regulators, and hypothetical proteins. Whereas the comparison of the BPD2 with 25525 genome exhibits the presence of a repeat region different than in the vaccine reference strains (Figure 2). These repeat regions carry flagellar genes, efflux genes, translation initiation factor, transcriptional regulators, ribosome binding protein, hypothetical proteins and genes involved in metabolism of *B. pertussis*. Such transposable DNA elements were regarded as the potent force in the evolution of bacteria 18.

The homologous recombination between copies of IS*481* has been attributed to genome reduction in *B. pertussis* which also suggests possible genome expansion by the same mechanism. Similarly, BPD1 and BPD2 have undergone genome reduction due to IS*481* comparable to vaccine strains 6229 and 25525. The lesser number of structural genes adds up to the potential of *B. pertussis* to be more virulent as it reduces the number of targets that are available for recognition by the human immune system 19.

Genomic plasticity in natural populations of *B. pertussis* is mainly through gene acquisition, loss, and genomic organization. Horizontal gene transfer (HGT) is one of the mechanisms responsible for genome evolution in *B. pertussis* 20. In this study, analysis of mobile genetic elements showed that BPD2 isolate had one intact phage region while no intact phages has been found in BPD1. Whereas genomic islands consisting of genes involved in carbohydrate, amino acid metabolism, membrane transport and transposases were identified in both the isolates. However, we did not observe CRISPR sequences, plasmids, acquired antibiotic resistance genes and mutation in 23S rRNA gene associated with macrolide resistance in *B. pertussis*.

### Phylogenomic relation between clinical strains and vaccine reference strains

**Figure 3:**
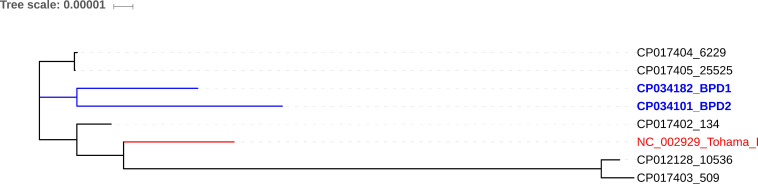
Core-genome based phylogenetic comparison of BPD1 and BPD2 Indian clinical strains with 134 (CP017402), 509 (CP017403), 10536 (CP012228), and Tohama I (NC_002929) vaccine reference strains.

Core-genome based phylogenetic analysis of the clinical strains and vaccine reference strains revealed that 6229 and 25525 were reported to be more closely related to BPD1 and BPD2 than the other vaccine strains of India 134 (CP017402), 509 (CP017403) and 10536 (CP012228) (Figure 3) 2, 21. This could be due to the switch between whole-cell and acellular vaccines. Interestingly, 134 was found to be closely related to the global reference strain Tohama I. Whereas 509 and 10536 showed high genetic distance to all other strains.

### Toxin and other virulence genes

The isolates were analysed for the presence of major virulence and toxin genes including pertactin (*prn*)*,* fimbriae (*fim*2*, fiim*3), filamentous (*FHA*) and pertussis toxin (*dnt, cyaA, ptx*A, *ptx*B, *ptx*C, *ptx*D, *ptx*E, *ptx*S). Both BPD1 and BPD2 isolates had *ptx*S toxin genes with all five subunits and carried other genes with no allelic variation compared to vaccine reference strains except *cya*A gene in BPD2 which had amino acid substitution of G552R and *ptx*B gene in both the isolates had substitution of G45S. This observation supports the hypothesis that pathogen adaptation is mainly through antigenic divergence between vaccine strains and circulating strains 17. Other genes identified include *vir*B2, B3, B6, B7, B8, B9, B10 and B11 (Table 1).

### Pathogen identities

Moreover, the PathogenFinder for BPD1 proteome families matched with 10 non-pathogenic families and 3 pathogenic families. This showed sequence similarity of 88%, 85.9% and 87.18% to the pathogenic families of *Burkholderia cenocepacia*,* Burkholderia mallei* and* Neisseria gonorrhoeae* respectively. Similar results were observed for BPD2.

## Conclusion

Utilizing the hybrid genome technology, we report the highly accurate complete genome sequences of *B. pertussis* Indian clinical strains. Comparison of clinical strains with vaccine reference strains of India revealed presence of existing and new insertional regions (~120 KB and ~180 KB) attributing to the genome degradation and expansion. Also, the resulting antigenic variation against the vaccine reference indicates that *B. pertussis* is evolving under vaccine driven selection. The impact of these events needs further investigation using a larger collection of clinical isolates. However, this comparative genome analysis will help to decipher more information on the genome evolution, virulence factors and their role in pathogenesis for the development of effective vaccine strategies in India.

## Figures and Tables

**Table 1:** Virulence genome characteristics of clinical *B. pertussis* from India

Strain	BPD1	BPD2
**Accession number**	CP034182	CP034101
~120 Kb Repeat insertion flanked by copies of IS*481* 1,240,892 to 1,359,872	√	-
~180 Kb Repeat insertion flanked by copies of IS*481* 2,186,762 to 2,372,309	-	√
**Virulence factors**		
Pertactin; *prn*	√	√
Dermonecrotic toxin	√	√
Pertussis toxin; ptxS1, ptxS2, ptxS3, ptxS4, ptxS5	√	√
Putative toxin; Toxin subunit PtxB/PtxC-related protein	√ ^#^, G45S	√ ^#^, G45S
Bifunctional adenylate cyclase toxin/ hemolysin CyaA	√	√, G552R
RTX toxins determinant A and related Ca2+-binding proteins/Cytolysin-adenylate cyclase	√	√
toxin-activating lysine-acyltransferase	√	√
toxin-antitoxin system CptAB antitoxin	√	√
type II toxin-antitoxin system HipA family toxin	√	√
type II toxin-antitoxin system HicB family antitoxin	√	√
type II toxin-antitoxin system HicA family toxin	√	√
type II toxin-antitoxin system RatA family toxin	√	√
type II toxin-antitoxin system MqsA family antitoxin	√	√
type II toxin-antitoxin system MqsR family toxin	√	√
Type III secretion proteins	√	√
Type IV secretion proteins	√	√
*vir*B2 - B11	√	√
Filamentous hemagglutinin *fha*B, *fha*C	√ ^*^, G280S, A281T	√

^*^ *fha*B; ^#^ *ptx*B.
